# “Nothing works without the doctor:” Physicians’ perception of clinical decision-making and artificial intelligence

**DOI:** 10.3389/fmed.2022.1016366

**Published:** 2022-12-20

**Authors:** David Samhammer, Roland Roller, Patrik Hummel, Bilgin Osmanodja, Aljoscha Burchardt, Manuel Mayrdorfer, Wiebke Duettmann, Peter Dabrock

**Affiliations:** ^1^Institute for Systematic Theology II (Ethics), Friedrich-Alexander-Universität Erlangen-Nürnberg (FAU), Erlangen, Germany; ^2^German Research Center for Artificial Intelligence (DFKI), Berlin, Germany; ^3^Department of Nephrology and Medical Intensive Care, Charité—Universitätsmedizin Berlin, Corporate Member of Freie Universität Berlin, Humboldt-Universität zu Berlin, and Berlin Institute of Health, Berlin, Germany; ^4^Department of Industrial Engineering and Innovation Sciences, Philosophy and Ethics Group, TU Eindhoven, Eindhoven, Netherlands; ^5^Division of Nephrology and Dialysis, Department of Internal Medicine III, Medical University of Vienna, Vienna, Austria

**Keywords:** artificial intelligence, physician, decision support, health, qualitative content analysis

## Abstract

**Introduction:**

Artificial intelligence–driven decision support systems (AI–DSS) have the potential to help physicians analyze data and facilitate the search for a correct diagnosis or suitable intervention. The potential of such systems is often emphasized. However, implementation in clinical practice deserves continuous attention. This article aims to shed light on the needs and challenges arising from the use of AI-DSS from physicians’ perspectives.

**Methods:**

The basis for this study is a qualitative content analysis of expert interviews with experienced nephrologists after testing an AI-DSS in a straightforward usage scenario.

**Results:**

The results provide insights on the basics of clinical decision-making, expected challenges when using AI-DSS as well as a reflection on the test run.

**Discussion:**

While we can confirm the somewhat expectable demand for better explainability and control, other insights highlight the need to uphold classical strengths of the medical profession when using AI-DSS as well as the importance of broadening the view of AI-related challenges to the clinical environment, especially during treatment. Our results stress the necessity for adjusting AI-DSS to shared decision-making. We conclude that explainability must be context-specific while fostering meaningful interaction with the systems available.

## Introduction

The ever-increasing performance of computers and the availability of vast amounts of data have led to an immense progress in the field of Artificial Intelligence (AI) and machine learning in recent years (1). In the field of medicine, Artificial Intelligence–driven Decision Support Systems (AI-DSS) are currently receiving much attention. These AI applications based on machine learning or deep learning methods offer great potential to support clinical decision-making (2, 3), for example by reducing the workload of medical staff (4).

The impact of AI-DSS on clinical decision-making can already be observed in a number of settings. In image recognition, systems can already interpret images more accurately in some respects than medical experts (4). In oncology, *Watson for Oncology* suggests therapy options for cancer patients from the medical literature and is intended to serve as an alternative to the established method of the interdisciplinary tumor conference (5). For remote health assessments, systems provide factual diagnostic suggestions in the form of symptom checkers (6). Of particular interest for the following study is the possibility of risk assessment, i.e., making statements about the current or future health status of patients (7).

Decision support is of course far from new in clinical decision-making. For decades, efforts have intensified to systematically consider and operationalize available evidence into clinical practice guidelines (8), hereby “packaging evidence and present recommendations to healthcare decision makers” (9). A novel, distinctive challenge raised by AI-DSS is the proliferation of available data points and the increasing computational complexity of available applications to process them. On the one hand, these can enhance diagnostic and predictive power, thereby enhancing clinical utility. On the other hand, one exemplary challenge is that not only for the treating physician, but even for designers of the decision support tool, these developments lead to a considerable increase in epistemic opacity about how and why a given output is provided (10–12).

This creates challenges beyond the performance of an AI-DSS. While the importance of trust, the attribution of responsibility, and transparency has been much debated in the literature (10), there is still a lack of knowledge about the needs of physicians in their daily work concerning these issues (13). The hypothesis prompting the present study is that the deployment of AI-DSS can be seen as a socio-technical challenge, which means that the available systems must always meet the needs of the users and the environment in which it will be deployed (14). Thus it is important to take into account the given processes and workflows in which clinical decision-making takes place (15). In line with this hypothesis, there is a growing realization that empirical insights into the actual application of AI-DSS are of great importance. Qualitative analysis can take a closer look at the role of healthcare professionals when applying AI-DSS (16–18), especially in answering the question of physicians’ involvement in the development and implementation of AI-DSS (19, 20).

The aim of the present study is to empirically explore the attitudes and perspectives of physicians toward a particular, novel, machine-learning-based AI-DSS use case in nephrology. We conducted a qualitative study to investigate the empirical question of how physicians experience operating with AI-DSS implementations in view of the outlined challenges. Our study seeks to identify the issues they perceive and anticipate, and the strategies they envision to be helpful in developing relevant AI-DSS further.

## Materials and methods

The empirical data consists of 14 semi-structured expert interviews conducted with physicians experienced in kidney transplant care. The interviews were performed after an experiment during which the physicians tested an experimental AI-DSS intended to assist in the risk assessment of patients after kidney transplantation. The system tested is based on routine clinical data from around 1,500 patients and more than 100,000 data points and makes predictions for the risk of infection and graft loss in the next 90 days. Internal validation showed AUC-ROC values of 0.83 for rejection and 0.95 for graft failure, which were promising results. To compare the performance of nephrologists with and without the system, a reader study was performed: the physicians were asked to make predictions for the risk of rejection and graft loss in the next 90 days, first without and in a second part with the AI system’s recommendations. First, the reader study shows that physicians’ predictions align with those of the AI-DSS. However, performance does not improve (AUC-ROC; 0.6413 vs. 0.6314 for rejection; 0.8072 vs. 0.7778 for graft failure) (21). Even though the system seems promising and outperformed physicians on the tasks, additional questions arise of whether such a system would add any value to clinical decision-making, how it should be implemented, and which chances and concerns are seen by physicians. This led to the decision to complement the experiment with a subsequent qualitative study.

### Expert interviews as a method for data collection

As the name of expert interviews implies, the interviewees are not interested in their totality, but in their specific role as experts which already indicates a narrowly defined social context. The aim of the method is to gain access to the knowledge of the experts, which includes their experiences as well as the specific rules and structures of the social environment in which they interact (22). For this reason, it is recommended that the interviews not only be conducted exploratively, as is quite common with other qualitative methods, e.g., in narrative interviews. In order to address an expert, it is advisable to adjust the questions to the specific context (22).

The principal aim of qualitative social research is to describe routines, patterns of interpretation, and structural features of social realities by evaluating material obtained from communication or interaction processes (23). Therefore, expert interviews also offer the possibility of approaching the collected material inductively (24). Even though the interview guide may narrow the framework, expert interviews still offer space for what is called the “exploration of the unknown” (22).

### Data collection

During the collection of the interviews, we kept in mind the ambivalent nature of the expert interview. The social context in which the interviews took place is narrowly defined by the clinical environment and the physicians as part of the medical profession. Therefore, the interview guide was based on theoretical assumptions regarding this setting. Initially, our research interest was to learn how the physicians perceived the AI-DSS in the previous experiment. In addition, the interview guide included questions concerning trust, transparency, and responsibility which are much discussed normative challenges in the literature on AI-DSS (10). Our goal was to give physicians the opportunity to highlight further issues that they see related to the use of AI-DSS in clinical decision-making. Therefore, we used open questions, so that the physicians could also incorporate their own thoughts and modes of expression in the interview.

We included all senior physicians with experience in kidney transplantation at our institution, who were willing to participate and not involved in study conception or conduction. Since a total of seven senior physicians participated in the study, we decided to include the same number of junior physicians, resulting in a total of 14 participants. Even though we aimed for a balanced gender distribution of the study participants, no focus is placed on this in the analysis, since gender is not relevant when working with AI-DSS.

### Qualitative content analysis in data evaluation

**FIGURE 1:**
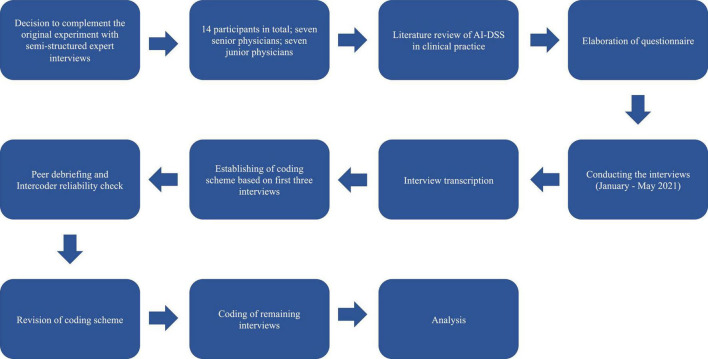
Process model of the survey according to Qualitative Content Analysis by Philipp Mayring (25).

The entire process of analyzing the expert interviews is based on Qualitative Content Analysis according to Mayring (25). The method was chosen because it offers both the possibility to address the material from previously developed theoretical perspectives, as well as to approach the interviews inductively (25). Thus, this method is suitable for our undertaking, without neglecting the physicians’ perceived issues. The goal of qualitative content analysis is the interpretative assignment of text passages in the collected material, as well as their systematization. Mayring is keen to ensure that qualitative research is rule-governed. For this reason, he recommends defining a process model (see Figure 1) from the beginning, in which a plan is drawn up for how the material is to be approached. At the center of the analysis lies the coding of the material. Finally, the central content should be reduced to a manageable level (25).

### Data analysis

After interview transcription, analysis was carried out with the help of *Atlas.Ti* coding software. Taking into account the theoretical assumptions mentioned above, three interviews were coded by a single researcher. The resulting codes were then edited and combined into a first coding scheme. Therefore, it was important at this point to meet the requirement of intersubjective verification. Thus, we decided to conduct an intercoder reliability check as well as several peer debriefings. These two methods are often used to check the validity of results obtained through qualitative research (26). The intercoder reliability check was conducted by an additional researcher analyzing the coding scheme of the first three interviews. To this end, the interim results were discussed in detail with other scientists. Subsequently, the results of the new schemes were compiled, incorporating feedback from the discussions, resulting in a coding scheme that was applied to the remaining interviews.

For the interpretation, the individual subcodes were once again examined more closely by two researchers. During the coding process, memos and comments were written to point out text passages that seemed particularly relevant and meaningful. This procedure was inspired by methods used in Grounded Theory (27). However, a final interpretation was conducted only after coding had concluded and revealed the following results. As the interviews were conducted in German, the quotes presented have been translated into English. The translation was conducted by the authors. To improve the flow of reading, the quotations have been smoothed out. Multiple discussions within the interdisciplinary research team, and particularly between the two researchers who conducted the interviews, ensured that the meaning of the quotes used was not altered during this process.

## Results

The numbers given in the results are quotes that can be found in the Supplementary material. A total of nine themes are presented in three main subsections.

The first subsection focuses on trust, responsibility, and the accessibility of information that is the basis of clinical decision-making. Since trust and responsibility are relational concepts, the importance of interpersonal exchange is also considered (see section “Basics of clinical decision-making”). The second chapter focuses on physicians’ reports about challenges they anticipate in the implementation of AI-DSS. While they see opportunities, challenges include the risk of losing experience and the need to explain the results of an AI-DSS to patients. In addition, references to transparency in the form of explainability emerged (see section “Challenges concerning artificial intelligence-driven decision support systems”). Finally, we describe physicians’ reflection on the interaction with the AI-DSS, the future use of AI-DSS, and their suggestions for improvement (see section “Reflection of the experiment”).

### Basics of clinical decision-making

In the interviews, trustworthy decision-making is portrayed as requiring evidence and experience. In addition, the exchange with the patient, colleagues, superiors, and nursing is emphasized. These topics are particularly important in connection with responsibility, which the interviewed physicians locate above all in relation to inform the patient adequately.

#### Evidence and experience

In interviewees’ views on what trustworthy decisions with AI-DSS involves, a contradiction between evidence-based and eminence-based decision-making initially becomes apparent. First, the physicians understand trustworthy decision-making as reflecting present structures and standards in medicine (Q1). For the sake of patient safety, any chosen treatment must rely on prospective clinical studies which validate its effectiveness (Q2, Q3). In other words, decisions should be evidence-based. According to the interviewees, however, the ideal of evidence-based medicine cannot always be attained in everyday clinical practice (Q4), e.g., because of imperfect available evidence. In such cases, physicians talk about the importance of eminence-based decisions where human characteristics such as experience and intuition are considered important (Q5, Q6):

I think, clinical experience plays a big role, whereas it’s probably not, nah, clinical experience just plays a role, I think personally. We’re still working with people, and I think someone who is a very experienced clinician does sometimes make decisions based on gut instinct actually. And I’ve seen it many times that the decisions were not wrong. And sometimes, sometimes, things are, if one rather prospectively, actively acts to exclude things yes, perhaps [is] more sensible and even if nothing comes out of it now but to minimize a risk or at least somehow prospectively, actively to act, sometimes [it is] perhaps better. Also in the sense of the patient (Q7).

At the beginning of this quote, after a short hesitation, the interviewee justifies her opinion about the importance of experience. She refers to situations where more experienced physicians have had to make decisions that were not causally justified. This is clarified once again by the term “gut,” which refers to an intuitive decision or one based on feelings. In the interviews, experience and intuition appear to be important for trustworthy decision-making and as an important quality for any physician.

#### Interpersonal exchange

According to the interviewees, sound decision-making is a process that involves a variety of perspectives.

[…] the patient, I, other disciplines, laboratory physician and radiology and a senior physician. And I think sometimes, of course, the other team, other assistants, who point out something, […] who maybe did the examination, the nursing, I don’t know, the patient tells the nursing staff something else again or they notice something again, which I didn’t notice. So there are more people (Q8).

The physician makes it clear that a decision initially takes place between her and the patient. However, this decision seems to be intermediated by several instances. The reason for this is the need for specialist knowledge, which is indicated by the mention of other disciplines and laboratory medicine. Reference to the senior physician also indicates a hierarchical element. Particularly worth mentioning at this point is the nursing staff, who are consulted in view of their distinctive communicative relationship with patients. This diversity of perspectives in clinical decision-making is observed throughout the interviews (Q9–Q13).

#### Responsibility

The interviewees share the view that responsibility for decisions in everyday clinical practice lies with the attending physician (Q14–Q16). Thus, although patients are part of the decision-making process, physicians as experts should be providers of relevant knowledge. The existence of the physician-patient relationship is seen as giving rise to a sense of responsibility. Thus, their tasks include deciding how to communicate a diagnosis and a possible treatment (Q17). This includes taking a medical history, evaluating relevant parameters, and awareness of the patients’ personal situation (Q18). Each parameter from examinations and laboratory values can be reflected in the recommended therapy, but only through the conscious decision of the physician (Q19).

### Challenges concerning artificial intelligence-driven decision support systems

Physicians describe a need to incorporate AI-DSS into existing decision-making processes. They also express a certain unease about the use of AI-DSS potentially preventing critical thinking and acquisition of experience. As particular important appears the presented description of the physician-patient relationship. It is mentioned that using AI-DSS does not absolve the physicians from explaining the results of the system to the patient. At this point a need for transparency and explainability becomes apparent.

#### Opportunities and risks

As one of the perceived benefits of AI-DSS, it was stated:

That something objective is added. So that there is a lot of interaction with the patient and subjective assessments. And that one/that such a system, yes, like [.] an additional objective further arm, as if someone sits beside one (Q20).

The fact that, in addition to the interaction with patients and the patient’s own assessment, objective facts are used for diagnosis initially supports the classification of AI-DSS as a further parameter. However, the quote attributes even more to the AI-DSS: it creates the image of another person in the room which has the special ability to elevate objectivity.

This kind of humanization is also found in other quotations. For example, by describing an AI-DSS as an observer who looks over the shoulder of the treating physician (Q21), or by attributing conflict-resolving abilities to an AI-DSS (Q22). All of this appears in the interviews to be in connection with a great need among the interviewed physicians to receive confirmation and support in decision-making or to have an AI-DSS as an additional safety net (Q23–Q26).

If it is suspected that the need for support is becoming too great, a skeptical attitude is sometimes apparent in the interviews. Although it is assumed that the use of an AI-DSS will lead to a more critical attitude toward one’s own decision-making (Q27), for others the danger of feeling confirmed too quickly is highlighted.

I think the concern is always a little bit that also young colleagues quickly fall back on artificial intelligence and don’t shape their own instinct that much. And so that’s why I think/So for me, I would do it in a way that, yeah, I see that as confirmation or incentive or further input but try to stay with myself in this whole decision-making process. It’s a nice, yeah, on-top thing, but shouldn’t become the base, I think (Q28).

This quote makes the attitude in question visible. While the usage of an AI-DSS can lead to ease in the decision-making process, it could prevent the development of a clinical “instinct.” For that, especially less experienced physicians should be able to make their decisions without additional help and thus gain important experience.

#### Physician-patient relationship

Most of the interviewed physicians highlighted the importance of the direct encounter with patients as background condition for the potential deployment of any AI-DSS. According to them, this first impression already has an immense influence on the subsequent treatment (Q29). It is described how important it is to take patients seriously (Q30), to get to know them (Q31), to listen to their needs (Q32), and not to form an opinion in advance based solely on laboratory results (Q33). This relationship is important not only to obtain information relevant to the diagnosis but also to involve patients in decision-making according to their circumstances (Q34). It is important that each patient is considered as an individual case (Q35). Interaction with patients therefore differs greatly and it is necessary to obtain an overall picture of the individual condition (Q36). It is outlined that the patient also must trust the physician (Q37), because a therapy without the willingness of the patients is assumed to have little chance of success.

I am a counsellor who tries to get the best out of the patient and the final decision-making authority lies with the patient, because if he does not decide this himself, any therapy would probably be without success (Q38).

Thus, treatment is not only described as knowing relevant data of the patient but meeting her and involving her in the decision-making process (Q39). The challenge associated with the use of AI-DSS is that physicians must explain the use of the systems and the results to patients in a way they can understand in order to meet this requirement. Therefore, physicians must still be able to justify why a particular system is used and explain how it arrives at its result (Q40).

#### Transparency and explainability

The interviewed physicians mention that an AI-DSS should always have a certain degree of transparency (Q41). However, transparency does not seem to relate to the claim to enable full disclosure of the so-called “black box.” Physicians show awareness that it is a characteristic of many relevant forms of AI that not every step of the analysis can be made completely comprehensible (Q42). Instead, the interviewees underlined the need for establishing the efficacy of an AI-DSS. Moreover, system outputs should be reasonably explainable to the treating physician.

For instance, prospective clinical studies should ensure that the system’s data evaluation is not biased against certain groups of people (Q43). In all respects, a system still needs to prove reliable and must be implemented gradually in everyday clinical practice (Q44). For this, it should be explainable. Even if a system is based on sound evidence, this does not absolve the treating physicians from their communicative tasks in the physician-patient relationship. Presenting patients only with bare figures would appear untrustworthy (Q45). Rather, in the interviews, physicians are clearly assigned to the task of simplifying certain results to make them as comprehensible as possible for patients, depending on their individual needs (Q46).

### Reflection of the experiment

Physicians differed in terms of how they considered the system in their decision-making. Some physicians took the given recommendations as a departure point from which to arrive at their own judgment. Nevertheless, a large proportion of physicians preferred to first make their own assessment in its entirety and then use the system’s recommendation to critically question it. It has also been observed that when the rationale for the recommendation of the system could no longer be understood, a distancing took place. In these cases, the physicians often reverted to their own assessment. Systems like the tested AI-DSS are not expected to take over decision-making in the clinic soon. Physicians’ responsibility for a decision is thus seen as unlikely to be crowded out by the system, according to the physicians. Following on from the experiment, interviewees provide suggestions for improving AI-DSS in general and the tested system in particular.

#### Use of the tested artificial intelligence-driven decision support system

Overall, the system was used in two different ways. For example, one physician used the given risk prediction and considered it in the decision-making process from the beginning:

[…] it immediately gives me a direction in which I think. I am or we are all somehow always so professionally suspicious and I have the one, also now because that was the first time to experience this application there, now not immediately a hundred percent given myself to it. But it gives me immediately a trend and then relatively quickly if I already start to research then somehow, it actually always was confirmed. So I found as I said, it was then somehow a work relief (Q47).

In this case, the physician checks the direction that the system has given. It appears important for her to keep a critical attitude. This can be concluded from the statement that, according to the physician’s perception, a certain basic distrust is part of the medical profession. According to this, it would be inappropriate to simply accept the recommendation of an AI-DSS, at least not as long as such a system is new, i.e., has not yet proven reliable in trials and practice. Nevertheless, despite the need for a critical attitude, the reduction of the workload that the use of the system has brought is emphasized.

Another physician used the system in the experiment as a second opinion. The system was merely consulted at the end of the decision-making process to critically question her own assessment. Therefore, it took additional work for herself to use the given risk prediction:

I think I always tried to look first myself and then to ask the AI again whether I had overlooked any of the points that the model found important and so on. And have quasi-tried to get an unbiased view on it first and then again, again to let me support so to speak, because otherwise one becomes so lazy in thinking (Q48).

The quote clearly shows how the system is taken as an occasion to review the physician’s own assessment. In the beginning, she describes not having used the prediction. Her initial decision is independent of the AI-DSS and only subsequently compared with it. The rationale for the procedure is particularly interesting. The physician emphasizes the concern of becoming “lazy in thinking” if an AI tool is consulted prior to her own approach to the specific case. This suggests that there is a certain reservation about making decision-making too easy in advance.

Other interviewees stated that the willingness to accept the result of the system also depends on the extent to which it coincides with one’s own thoughts about the specific case. Confirmation, for example, led to a reduction of distrust (Q49), whereas differences with the system triggered uncertainty. In most cases, uncertainties were used to critically question one’s own assessment (Q50). When the difference could not be understood, however, some physicians quickly revert entirely to their own assessment:

So if I now, so if I was of the same opinion then I went along with it a bit, but if it was, completely, if I found it completely absurd, then I simply ignored it (Q51).

This quote summarizes well the difference it makes whether or not the system provides assessments that cohere with the physician’s assessment. Ignoring the system means to exclude it from the decision-making process. What is missing for the physician at this point is an understanding of why the AI-DSS arrives at such a differentiated assessment. The use of the word “absurd” indicates that she cannot explain it.

#### Expected impact on clinical decision-making

Reflecting on the experiment, the interviewees did not think that by using the system, they would hand over decisional authority to it (Q52). Physicians describe such a system as a parameter of its own that can be included in the evaluation along with other information for diagnosis. The system would then help to cope with large amounts of data (Q53, Q54) and thus make the decision-making process more efficient (Q55).

The responsibility for making decisions with an AI-DSS is still considered to be with the treating physician. For example, they suspect that not taking such responsibility for the decision of an AI-DSS could lead to possible diffusions or gaps where no one can be held accountable for an AI-supported decision anymore (Q56). Furthermore, handing over responsibility is associated with a loss of authority (Q57, Q58).

As an additional justification for the decision, such a system could help to carry the heavy burden of responsibility more easily (Q59). No further issues are identified. It is emphasized that AI-DSS can also be used in the future to develop a more critical attitude in the decision-making process (Q60). A fundamental change in the previously established structures in everyday clinical practice is not anticipated (Q61).

These points are in line with the way respondents would integrate such systems into their everyday work. It should, for example, prevent wrong decisions but not pre-empt decision-making (Q62). In the end, trust should be based on one’s own assessment, feeling, and intuition. One concern of the physicians is that the thinking person falls out of the decision-making process. The loss of human judgment is accompanied by a loss of the range of therapeutic options (Q63). Therefore, attention should always be paid to maintaining an attitude of critical thinking (Q64). For this, it must be prevented that AI-DSS stands in the way of the acquisition of expertise (Q65).

#### Suggestions for improvement

Taking into account the suggestions made by physicians to improve the use of AI-DSS, it appears that physicians want more information and further influence on the system. Several physicians would like additional explanations of how the system arrives at its assessment (Q66, Q67). In addition, the system’s assessment should not stand alone, but should be able to be linked to other programs (Q68). One specific suggestion was the possibility that the system would highlight further literature or research results to connect the prediction with further evidence instantly (Q69). Furthermore, the suggestion is made to implement mechanisms for users to influence which parameters are included in the analysis of the AI-DSS (Q70). To this end, the interviewed physicians want the system to provide traceable predictions.

## Discussion

Across the three themes that surfaced in the results of this study, a set of normative requirements for the use of AI-DSS can be inferred (see Table 1). Each of them latches onto and extends issues in current debates surrounding on AI in medicine. First, physicians’ claim to maintain expertise and autonomy in decision-making can be seen as an attitude associated with the medical profession. It is debatable whether this professional attitude provides good preconditions for the challenges associated with the deployment of AI-DSS (see section “Maintaining expertise and autonomy”). Furthermore, as the physicians’ decision-making does not take place in a vacuum it is important to consider the clinical environment. Questions including clinical organizational structures and how to shape the physician-patient relationship must be addressed in the discussion about AI-DSS (see section “Importance of the clinical environment”). Especially the responsibility to inform the patient refers to the need of thinking transparency and explainability not just technically, but to adapt it to the specific application context. It seems advisable to design the interaction between physicians and AI-DSS in such a way that it becomes possible to exert influence on it (see section “Explainability and meaningful interaction”).

**TABLE 1:** Themes in the results and inferred normative requirements.

Basics of clinical decision-making	Challenges concerning AI-DSS	Reflection of the experiment	Normative requirements
**Evidence and experience** Decisions must be evidence-based, but human characteristics like experience and intuition still play an important role when it comes to clinical decision-making.	**Opportunities and risks** Physicians anthropomorphize AI-DSS. That suggests the need to receive conformation and support. This support should not prevent physicians from making their own experiences.	**Use of the tested AI-DSS** The use of the tested AI-DSS took place with a critical distance. The physicians often took additional steps to verify the accuracy of the system’s results. Uncertainties led to a quick resort to one’s own assessment.	**Maintaining expertise and autonomy**
**Interpersonal exchange** Many perspectives must be included in the decision-making process. In addition to patient communication and consultation with the senior physicians, laboratory medicine and nursing staff are particularly important.	**Physician-patient relationship** The relation with patients is seen in the individual encounter. AI-DSS do not relieve physicians from the task of communicating the results to patients in a way they can understand.	**Expected impact on clinical decision-making** Physicians do not expect the use of AI-DSS to change clinical routines and responsibilities. Rather, AI-DSS appear as a new parameter that can be embedded in existing structures of decision-making.	**Roles in the clinical environment**
**Responsibility** Responsibility lies in collecting all relevant information about the patient but also informing the patient in an adequate way.	**Transparency and explainability** Transparency should partly be reached by providing evidence for an AI-DSS. Explainability is needed for physicians to understand the given results.	**Suggestions for improvement** Physicians emphasize the importance of influencing the recommendations from an AI-DSS and give creative suggestions for it.	**Promoting explainability and meaningful interaction**

### Maintaining expertise and autonomy

Our results show that interviewees feel responsible during the decision-making process even when using AI-DSS. They had a critical distance to the tested AI-DSS and emphasize that usage should not entail a loss of expertise. In line with this, they sought to retain decision-making autonomy. Physicians’ claim to maintain expertise and autonomy in decision-making reflect a classical understanding of the medical profession. Typical features of a formal profession include an academic education, a high degree of expert knowledge, a commitment to central social norms and values together with a high degree of autonomy (28). These characteristics should ensure that professionals are able to apply their expertise to individual patients and their particularities (28).

In principle, these characteristics of the medical profession, including its fiduciary duties to patients, could be promising sources for guiding and fostering trust in the development and deployment of AI-DSS in clinical practice (29). This being said, it is not obvious whether this classical understanding can be maintained when considering AI-DSS. As Noordegraaf (30) points out, the hallmarks of professionalism as such are being challenged. New technologies such as AI-DSS have a direct impact on physicians’ workflow, thereby possibly heralding a shift in competencies (30) and potentially transforming the constitutive characteristics of a given profession. In this sense, Noordegraaf offers a suggestion to rethink how professions may be reimagined in the light of the challenges they face: attributions such as expertise and autonomy must be considered in a relational manner. He claims that the distinctive features of the medical profession currently do not lie in mere formal characteristics but in the interconnectedness with other social practices.

Professionals not only act, but they also have to interact with many others and take performative action: how they do things, how they relate to client experiences, how they learn, how they deviate from standards, and how they appear trustworthy, that determines whether they are seen as “professionals” [(30): 209].

We thus suggest that the expertise and autonomy that interviewees’ upheld across the themes of the results should not solely be conceived of in terms of formal characteristics but also in terms of the roles and relational characteristics of the medical professional. For a given AI-DSS, it should be assessed whether it promotes or inhibits fulfillment of these relational features.

### Importance of the clinical environment

The importance of organizational structures for AI-DSS is a highly neglected area of research when brought to the discussion of AI-DSS (31). Our results indicate a need of AI-DSS users for a variety of interactions within routine communication with senior physicians, colleagues, laboratory physicians, and nursing staff. All these interactions e.g., at what point a junior physician consults a senior physician or the cooperation of different departments in the clinic pertain to the respective institution’s organizational structures. To ensure a beneficial deployment of an AI-DSS, a well-functioning interplay between the users, the available technology, and the organization is necessary (31). Since the physicians’ decision-making does not take place in a vacuum, any assessment of an actual implementation of clinical AI-DSS will thus need to be based at least in part on information about the institution’s organizational structures.

A simple but consistent demand flowing from the results is that the introduction of AI-DSS into everyday clinical practice should not inhibit or even prevent acquisition of experience and engagement in interpersonal interaction. Neither should it crowd out the patient’s role in the decision-making process. The concept of shared decision-making (SDM) is often considered as the optimal way to involve the patient in the treatment (32) and is also becoming more and more important in the discussion about AI-DSS. The goal of SDM is to provide the patient with the maximum amount of appropriate information, to actively consider the patient’s needs in decision-making, and to strengthen the physician-patient relationship (33). Our results underline that an AI-DSS in SDM should not constrain the physician-patient relationship, but provide relief to make an intensification of the relationship possible (34).

### Explainability and meaningful interaction

Interviewees’ remarks on explainability can be linked to the discussion around explainable AI (XAI). XAI is often mentioned as the key to deal with the problem of so-called “black box algorithms” and therefore deal with a lack of transparency and maintain trust and security (35). If an AI-DSS were explainable, users could in principle be enabled to interpret the system in a way that allows them to evaluate outputs, detect errors, and thus exercise control over the systems. Explainability can also bring its own difficulties. For example, a high demand is placed on the individual, who must not only understand the system to some extent, but also relate its use of it to her own goals (36).

XAI is thus not merely a technical challenge. There are always social components that affect how explanations are designed, e.g., for whom the explanations are made, what specific needs the users have, all the way to the question of what educational offerings are necessary to be able to understand the explanations given (37, 38).

The present study specifies that whether something counts as a useful explanation depends on how and for what physicians use the AI-DSS. For instance, since the outcome of an AI-DSS must be explained to patients, the interviewed physicians need an understanding of how the system arrives at its recommendations for this purpose. It is precisely these differences in background knowledge and presuppositions of different target audiences that need attention when pursuing explainability in AI-DSS.

Related to the issue of explainability, the physicians interviewed clearly indicate that they want to exert control over AI-DSS. This can be concluded from interviewees’ suggestions for improvement of the tested system, i.e., the desire to be able to influence system output. Such control presupposes certain forms of explainability. For example, the concept of meaningful human control (MHC) requires that users must be able to understand the impact an AI-DSS has in the context of the actual application (39). The physician must therefore not only be able to operate the AI-DSS, but also be able to assess in which cases the system should be used and how the recommendations of an AI-DSS should be interpreted in a context-specific manner. Careful attention should be devoted to how the interaction between an available AI-DSS and the user – in our case the physician – should be organized (40).

Holford (41) describes what context-specific use means for the question of how interaction with an AI system should be exercised. He claims that decisions, especially in fields that involve a lot of responsibility, should not be made by AI systems alone. Not because a system necessarily delivers worse decisions than a human, but because use of systems could actively prevent the essential involvement of humans and acquisition of experience and skills such as intuition. Without these, experts are unable to adequately assess new and complex situations. It is essential for every expert to live through certain situations to gain experience, which is then relevant to deal with problems that arise in other contexts (42). For Holford, an agent who bears responsibility in a situation in which an AI system is used must be able to exercise meaningful control over the system. Through a meaningful interaction with the system, the agent is directly involved in what is happening and thus can influence the situation in line with their expertise.

In this manner, it is primordial that future socio-technical designs be configured in a manner that avoids “operator hand-off” and subsequent “automation complacency” [(41): 8].

Expertise appears in this case as the ability to assess what is acutely important and relevant in a specific situation where decisions are required. In this sense, meaningful interaction is about enabling experience and expertise with, possibly even through, the use of AI-DSS, ensuring an understanding of a system’s outcomes, and enabling users to integrate systems into existing decision-making structures.

## Limitations

Like all empirical studies, the present one has certain limitations. In general, we believe that it makes sense to accompany and complement experiments in the medical field with qualitative methods of social research. However, it may also be that the experiment influenced the attitudes of the interviewed physicians. For example, our results show an interested attitude toward AI-DSS. Based on the present study, this should always be considered in the context of the experience of the previous experiment. In a different context, the attitude could be different.

Furthermore, by focusing on physicians, this study provides important insight into the challenges of clinical decision-making with AI-DSS. For the discussion on the implementation of AI-DSS in clinical practice, it is also important to interview all health care professionals as well as patients and developers to include their impressions, experiences, and opinions in the overall discussion.

## Conclusion

With the progress of AI-DSS performance, the question of how to implement relevant systems in practice becomes more central. The purpose of this study was to explore physicians’ perspectives after testing an AI-DSS for predicting risks in kidney transplant care. The results point to issues concerning the basics of clinical decision-making, expected challenges associated with AI-DSS, and specific considerations about the experiment in which the novel AI-DSS was tested. In general, the findings indicate a positive attitude toward AI-DSS. Concerns include a potential loss of expertise and autonomy. Of high importance is the clinical environment in which AI decision-support as well as patient involvement takes place. This is in line with the desire for further means of establishing explainability and control.

These findings resonate with current debates on AI in medicine. The question of how to implement AI-DSS in current organizational structures as well as the importance of ensuring shared decision-making remain equally central. Explainability must be context-specific and AI-DSS designed to ensure meaningful interaction. In this sense, expertise and autonomy of the treating physicians may be maintained when implementing AI-DSS. We hope the present study supplements conceptual discussions surrounding AI-DSS in medicine with empirical evidence and provides a starting point for further research.

## Data availability statement

The original contributions presented in this study are included in the article/Supplementary material. Further inquiries can be directed to the corresponding author.

## Ethics statement

Written informed consent was obtained from the individuals for the publication of any potentially identifiable data included in this article. It was ensured that the data did not allow any conclusions to be drawn about the individuals.

## Author contributions

RR, PH, BO, AB, MM, and WD contributed to the conception and design of the study. PH and BO executed the study and performed interviews. DS, RR, BO, AB, and PD performed the data analysis. DS wrote the manuscript. All authors contributed to the manuscript revision and read and approved the submitted version.

## References

[1] Coeckelbergh M. *AI Ethics.* Cambridge, MA: MIT Press (2020).

[2] Shafi I Ansari S Din S Jeon G Paul A. Artificial neural networks as clinical decision support systems. *Concurr Comput Pract Exp.* (2021) 33:e6342. 10.1002/cpe.6342

[3] Shailaja K Seetharamulu B Jabbar MA. Machine learning in healthcare: a review. *Paper presented at the 2018 Second International Conference on Electronics, Communication and Aerospace Technology (ICECA).* Coimbatore: (2018). p. 910–4. 10.1109/ICECA.2018.8474918

[4] Topol E. High-performance medicine: the convergence of human and artificial intelligence. *Nat Med.* (2019) 25:44–56. 10.1038/s41591-018-0300-7 30617339

[5] Jie Z Zhiying Z Li L. A meta-analysis of Watson for oncology in clinical application. *Sci Rep.* (2021) 11:5792. 10.1038/s41598-021-84973-5 33707577 PMC7952578

[6] Chambers D Cantrell AJ Johnson M Preston L Baxter SK Booth A Digital and online symptom checkers and health assessment/triage services for urgent health problems: systematic review. *BMJ Open.* (2019) 9:e027743. 10.1136/bmjopen-2018-027743 31375610 PMC6688675

[7] de Hond AAH Leeuwenberg AM Hooft L Kant IMJ Nijman SWJ van Os HJA Guidelines and quality criteria for artificial intelligence-based prediction models in healthcare: a scoping review. *NPJ Digit Med.* (2022) 5:1–13. 10.1038/s41746-021-00549-7 35013569 PMC8748878

[8] Kredo T Bernhardsson S Machingaidze S Young T Louw Q Ochodo E Guide to clinical practice guidelines: the current state of play. *Int J Qual Health Care.* (2016) 28:122–8. 10.1093/intqhc/mzv115 26796486 PMC4767049

[9] Treweek S Oxman AD Alderson P Bossuyt PM Brandt L Brożek J Developing and evaluating communication strategies to support informed decisions and practice based on evidence (DECIDE): protocol and preliminary results. *Implement Sci.* (2013) 8:6. 10.1186/1748-5908-8-6 23302501 PMC3553065

[10] Braun M Hummel P Beck S Dabrock P. Primer on an ethics of AI-based decision support systems in the clinic. *J Med Ethics.* (2021) 47:e3–3. 10.1136/medethics-2019-105860 32245804 PMC8639945

[11] Mittelstadt B Allo P Taddeo M Wachter S Floridi L. The ethics of algorithms: mapping the debate. *Big Data Soc.* (2016) 3:21. 10.1177/2053951716679679

[12] Tsamados A Aggarwal N Cowls J Morley J Roberts H Taddeo M The ethics of algorithms: key problems and solutions. *AI Soc.* (2022) 37:215–30. 10.1007/s00146-021-01154-8

[13] Antoniadi AM Du Y Guendouz Y Wei L Mazo C Becker BA Current challenges and future opportunities for XAI in machine learning-based clinical decision support systems: a systematic review. *Appl Sci.* (2021) 11:5088. 10.3390/app11115088

[14] Baxter G Sommerville I. Socio-technical systems: from design methods to systems engineering. *Interact Comput.* (2011) 23:4–17. 10.1016/j.intcom.2010.07.003

[15] Petitgand C Motulsky A Denis J-L Gis C. Investigating the barriers to Physician adoption of an artificial intelligence- based decision support system in emergency care: an interpretative qualitative study. *Digit Pers Health Med.* (2020) 270:1001–5. 10.3233/SHTI200312 32570532

[16] Ferretti A Schneider M Blasimme A. Machine learning in medicine: opening the new data protection black box. *Eur Data Prot Law Rev.* (2018) 4:320–32.

[17] Jongsma KR Bekker MN Haitjema S Bredenoord AL. How digital health affects the patient-physician relationship: an empirical-ethics study into the perspectives and experiences in obstetric care. *Pregnancy Hypertens.* (2021) 25:81–6. 10.1016/j.preghy.2021.05.017 34090186

[18] Taber P Weir C Butler JM Graber CJ Jones MM Madaras-Kelly K Social dynamics of a population-level dashboard for antimicrobial stewardship: a qualitative analysis. *Am J Infect Control.* (2021) 49:862–7. 10.1016/j.ajic.2021.01.015 33515622 PMC8991288

[19] Schaaf J Prokosch H-U Boeker M Schaefer J Vasseur J Storf H Interviews with experts in rare diseases for the development of clinical decision support system software a qualitative study. *BMC Med Inform Decis Mak.* (2020) 20:230. 020 01254 3 10.1186/s12911 32938448 PMC7493382

[20] Schwartz JM Moy AJ Rossetti SC Elhadad N Cato KD. Clinician involvement in research on machine learning–based predictive clinical decision support for the hospital setting: a scoping review. *J Am Med Inform Assoc.* (2021) 28:653–63. 10.1093/jamia/ocaa296 33325504 PMC7936403

[21] Roller R Mayrdorfer M Duettmann W Naik MG Schmidt D Halleck F Evaluation of a clinical decision support system for detection of patients at risk after kidney transplantation. *Front Public Health.* (2022) 10:979448. 10.3389/fpubh.2022.979448 36388342 PMC9641169

[22] Liebold R Trinczek R. Experteninterview. In: Kühl S Strodtholz P Taffertshofer A editors. *Handbuch Methoden der Organisationsforschung: Quantitative und Qualitative Methoden.* Wiesbaden: VS Verlag für Sozialwissenschaften (2009). p. 32–56. 3 531-91570 8_3 10.1007/978

[23] Flick U Kardorff E Steinke I. Was ist qualitative forschung? Einleitung und überblick. In: Flick U Kardorff E Steinke I editors. *Qualitative Forschung. Ein Handbusch.* Hamburg: Rowohlt Taschenbuch Verlag (2000).

[24] Meuser M Nagel U. Das Experteninterview–konzeptionelle Grundlagen und methodische Anlage. In: Pickel S Pickel G Lauth H-J Jahn D editors. *Methoden der vergleichenden Politik- und Sozialwissenschaft: Neue Entwicklungen und Anwendungen.* Wiesbaden: VS Verlag für Sozialwissenschaften (2009). p. 465–79. 10.1007/978-3-531-91826-6_23

[25] Mayring P. *Qualitative Inhaltsanalyse: Grundlagen und Techniken.* Weinheim: Beltz (2015).

[26] Misoch S. *Qualitative Interviews.* Berlin: De Gruyter Oldenbourg (2019).

[27] Böhm A. Theoretisches codieren: textanalyse in der grounded theory. In: Flick U Kardorff E Steinke I editors. *Qualitative Forschung. Ein Handbuch.* Hamburg: Rowohlt Taschenbuch Verlag (2000).

[28] Abbott AD. *The System of Professions: An Essay on the Division of Expert Labor.* Chicago, IL: University of Chicago Press (1988).

[29] Mittelstadt B. Principles alone cannot guarantee ethical AI. *Nat Mach Intell.* (2019) 1:501–7. 10.1038/s42256-019-0114-4

[30] Noordegraaf M. Protective or connective professionalism? How connected professionals can (still) act as autonomous and authoritative experts. *J Profess Organ.* (2020) 7:205–23. 10.1093/jpo/joaa011

[31] Herrmann T Pfeiffer S. Keeping the organization in the loop: a socio-technical extension of human-centered artificial intelligence. *AI Soc.* (2022). 10.1007/s00146-022-01391-5

[32] While A. Shared decision-making. *Br J Commun Nurs.* (2019) 24:250–250. 10.12968/bjcn.2019.24.5.250 31059294

[33] Thomas EC Bass SB Siminoff LA. Beyond rationality: expanding the practice of shared decision making in modern medicine. *Soc Sci Med.* (2021) 277:113900. 10.1016/j.socscimed.2021.113900 33838448 PMC8119352

[34] Rahimi SA Cwintal M Huang Y Ghadiri P Grad R Poenaru D Application of artificial intelligence in shared decision making: scoping review. *JMIR Med Inform.* (2022) 10:e36199. 10.2196/36199 PMC9399841 35943793

[35] Holzinger A Kieseberg P Weippl E Tjoa AM. Current advances, trends and challenges of machine learning and knowledge extraction: from machine learning to explainable AI. In: Holzinger A Kieseberg P Tjoa AM Weippl E editors. *Machine Learning and Knowledge Extraction, Lecture Notes in Computer Science.* Cham: Springer International Publishing (2018). p. 1–8. 10.1007/978-3-319-99740-7_1

[36] König PD. Challenges in enabling user control over algorithm-based services. *AI Soc.* (2022). 10.1007/s00146-022-01395-1

[37] Asghari H Birner N Burchardt A Dicks D Faßbender J Feldhus N *What to Explain When Explaining is Difficult. An Interdisciplinary Primer on XAI and Meaningful Information in Automated Decision-Making.* Genève: Zenodo (2022). 10.5281/zenodo.6375784

[38] Ratti E Graves M. Explainable machine learning practices: opening another black box for reliable medical AI. *AI Ethics.* (2022) 2:801–14. 10.1007/s43681-022-00141-z

[39] Horowitz M Scharre P. *Meaningful Human Control in Weapon Systems: A Primer.* (2015). Available online at: https://www.cnas.org/publications/reports/meaningful-human-control-in-weapon-systems-a-primer (accessed November 29, 2022).

[40] de Sio FS Mecacci G Calvert S Heikoop D Hagenzieker M van Arem B. Realising meaningful human control over automated driving systems: a multidisciplinary approach. *Minds Mach.* (2022). 10.1007/s11023-022-09608-8 35915817 PMC9330947

[41] Holford WD. Design-for-responsible’ algorithmic decision-making systems: a question of ethical judgement and human meaningful control. *AI Ethics.* (2022) 2:827–36. 022 00144 w 10.1007/s43681

[42] Coeckelbergh M. Good healthcare is in the “how”: the quality of care, the role of machines, and the need for new skills. In: van Rysewyk SP Pontier M editors. *Machine Medical Ethics, Intelligent Systems, Control and Automation: Science and Engineering.* Cham: Springer International Publishing (2015). p. 33–47. 10.1007/978-3-319-08108-3_3

