# Supplementary material for: “Nothing works without the doctor:” Physicians’ perception of clinical decision-making and artificial intelligence
Source: Front Med (Lausanne). 2022 Dec 20;9:1016366. doi: 10.3389/fmed.2022.1016366 (PMC9807757; doi:10.3389/fmed.2022.1016366)
Supplement: Supplementary file 1 [file Data_Sheet_1.pdf]

“Nothing works without the doctor.” Physicians’ perception of clinical decision-making and artificial intelligence

Supplementary Material

Selected quotes from 14 semi-structured expert Interviews with junior and senior physicians.

Yes, perhaps it depends on how broadly you want to define it now, but, yes, there are these ideas of trust, but with medical devices there is perhaps more of a transition to the fact that there is also a very technical trust through the fact that there are certain standards that are ensured and structures exist that guarantee safety for patients and for the use of medical devices. And for all possible scenarios that somehow happen in a clinic [1, junior]
So in general I need a study that shows me that the tool brings added value, yes? For example, that it can prevent certain events or make the handling more effective. That it can better predict the critical event, faster, more autonomously. So it must/ needs practically, one study that brings me the proof of effectiveness. Then I start to believe. Yes, and actually it’s not just one study that is needed, it takes several studies. It needs multicentre systems established in other clinics. Then we start to believe that it will be of some use. Yes, that's just the way it is in medicine [2, senior]
Yes, […] if it predicts a probability of one hundred per cent and you have tested it in a study beforehand and it always came true one hundred per cent then at some point the trust is there. But/ Yes, you need a study beforehand that proves that. So you need proof that you can get involved in a black box [3, senior]
[…] I understand that some things are not always causally explainable. Or (..) whatever I believe, for, well, physicians, it is always nice to have a causal explanation, so that you can somehow imagine it pathophysiologically. But okay, we know that association is not causality. That is why a pure association, which is not causal, would also be/ It is the case with many things that they are associated but not causally linked, so that it is probably not always possible and I would also be satisfied if I knew there was a, well, coincidence, or something like that. But this one may not be causal, but it still helps me in predicting infections, or something like that [4, junior]
One can always assume some inaccuracy of the system and first of all assume that one's own estimations, which one makes, which are based on any estimations of a free text information or any more complex time courses, yes, so I would have now assumed that they are not necessarily taken into account by the system, so that one still, yes, now

trusts one's own feeling first of all or in a certain way whether that is right or not [5, junior]
Well, that is difficult. Many of my decisions are influenced by intuition and intuition is also knowledge. And most of the time, when I'm asked afterwards why I have decided this way, I can justify it [6, senior]
I think, clinical experience plays a big role, whereas it's probably not, nah, clinical experience just plays a role, I think personally. We're still working with people, and I think someone who is a very experienced clinician does sometimes make decisions based on gut instinct actually. And I've seen it many times that the decisions were not wrong. And sometimes, sometimes, things are, if one rather prospectively, actively acts to exclude things yes, perhaps [is] more sensible and even if nothing comes out of it now but to minimize a risk or at least somehow prospectively, actively to act, sometimes [it is] perhaps better. Also in the sense of the patient [7, senior]
[…] the patient, I, other disciplines, laboratory physician and radiology and a senior physician. And I think sometimes, of course, the other team, other assistants, who point out something, […] who maybe did the examination, the nursing, I don't know, the patient tells the nursing staff something else again or they notice something again, which I didn't notice. So there are more people [8, junior]
I ask my senior physician [9, junior]
It is based on the clinical impression, the first [impression] and the second after a long conversation. And often you verify it or compare it with the impression or experience of another person, the colleague [10, senior]
[…] you sometimes discuss certain decisions with your colleagues. [They] say, "Would you do it that way or not?" Or you try somehow to get a second opinion from experienced colleagues in difficult decisions. And this second opinion procedure, I think, is very important. [It is important] to discuss things. And I think as soon as you [have] a corrective or a problem is just discussed, this, I think, makes the decision sharper […]. That encourages you in the decision-making process. So I think the process is faster. The process is more precise, less wrong decisions, and in the end [there is] a better treatment [11, senior]
Okay, but partly also the care for example, so I'm currently in the emergency room, I always find this helpful, [because] they already have a lot of experience. Then [there are] of course with an X-ray finding or CT or the radiology, and also [other] departments that send me findings. And if I'm unsure or don't know exactly, I discuss it with colleagues or superiors [12, junior]

For the most part [I make the decision] independently. If somehow a problem, or if my expertise is not sufficient for this problem, I consult an expert who has either more or longer clinical experience. Or if the problem has perhaps not been sufficiently researched. I still consult colleagues who I know will provide helpful input or that it's then a joint decision, that at least you can bear it together if you actually do not have enough data for a good basis for decision-making. I involve the patient in the decision-making process. Because in the end he has to bear this decision. Or bear the consequences [13, senior]
Yes, I think as a physician you have a responsibility for the patients that you treat yourself. Yes, in the sense that you should take the greatest care of your patients [14, junior]
I have the responsibility for decisions even without the system. And with the system, too, because I use it [15, junior]
Well, I am responsible to my patients and I always bear the final responsibility. That is not borne by the AI. My decision is there. I then have to justify and/ or prove how I make it. [16, senior]
Yes (.) patient-centered, focused making of decisions, together with the patient, making therapy suggestions and then we implement them together. [17, senior]
I think that you should (.) do evidence-based medicine. It is extremely important, however, that you also take the patient on board, that you can also then carry out the personal wishes and needs. Otherwise, one (.) otherwise you do not need the doctor. [18, senior]
Because in the end I am still the person who communicates, explains, prescribes and orders this to the patients. Exactly. I'm actually still standing at the end of the flagpole because I am sitting in front of that person and have the conversation [19, senior]
That something objective is added. So that there is a lot of interaction with the patient and subjective assessments. And that one/ that such a system, yes, like [.] an additional objective further arm, as if someone sits beside one [20, senior]
It would probably be helpful if there was a second system looking over it in the form of a system and the AI as a second system. And then there are notifications that something could happen in the next few months or that things are going well or something. That you get a second feedback [21, junior]
[…] I think that especially when it comes to controversial cases or when different opinions arise, it would be very interesting to see what the independent observer actually says [22, senior]

[I]f AI can do a better job, whether someone is compliant or not, and whether it significantly increases the risk. Then it would also be a point to intervene earlier or to say: Look here, my artificial intelligence is warning me, or tells me that this is very strongly increased in the next few days, months. Maybe like having a whisperer, yes, who helps you with the decisions [23, senior]
[..] I would [use] it as a supporting element. […] I, as a physician, lay [a recommendation] down and say: look here how the computer, the machine says different things and compare. Like this, yes. That is an underpinning [24, junior]
[I] think it can help me to become a bit more confident in some decisions, because it somehow shows me more which algorithm I actually use to look at a patient. [...] if I continue in this way, then it supports me that I am somehow right and perhaps takes away a bit of the fear of being completely wrong because I have the values a bit more objectifiable than I have made them myself in my head or in my procedure [25, junior]
[…] I still believe that it has great potential to improve treatment. I am firmly convinced of that. And I also believe that it will be indispensable in the next few years. So I think it will be fundamental, it will be part of the therapy, because it just makes so much sense to make the treatment more efficient [26, senior]
I think in the end I would also be quite sceptical, but probably I would of course trust my thoughts also then, right? If I can't understand why such a programme can tell me something, I can't use it. But if it were on paper, of course I would have to think very critically and see where I might have overlooked something myself [27, senior]
I think the concern is always a little bit that also young colleagues quickly fall back on artificial intelligence and don't shape their own instinct that much. And so that's why I think/ So for me, I would do it in a way that, yeah, I see that as confirmation or incentive or further input but try to stay with myself in this whole decision-making process. It's a nice, yeah, on-top thing, but shouldn't become the base, I think [28, senior]
So I don't know how everybody else does it, but, um, you often have a first impression, that is a clinical one, of a patient or of a situation or something, and I actually rely on that. And I don't know if this emotion enters into this consideration, I am very much guided by it [29, senior]
[T]o take the patient with his complaints seriously and try to find solutions to problems for these complaints. And then to decide together with the patient, after reviewing the options, what the best therapy is [30, junior]
I think it's actually, you also have to know the patient. That means that for me, everything starts from the moment the patient enters through the door, right? And there

you can already get quite a lot of information, that is, about character, about stature, about the general condition, what you hear and see and so on. That's the first impression. Then of course comes the factual [31, senior]
[E]ven then you should do evidence-based medicine. It is extremely important that you also get the patient on board so that you can […] also do that. And you also listen to the personal wishes and needs. Otherwise, you don't need a doctor either [32, senior]
In principle, one should always look at the patient contact first. So [you should] try to give as little prior information to the patient as possible. One should not somehow say in advance [that something has occurred] due to some infection value or whatever. And in the end, one gets a picture, is he well, is he unwell and then subsequently laboratory parameter control and then finally make the decision [33, senior]
[I]t depends on the severity of the decision whether the patient is also involved or whether you just do it. With such profound decisions, of course, one will not only ask the patient, but also educate him or her in order to obtain consent. And otherwise, perhaps the family [34, junior]
Because I think that these are still totally individual cases and that you just can't generalise all patients like that and put them down in percentages, with probabilities and so on. But that there are always individual factors that come into play. And I believe that there are simply many different factors and that it is not necessarily possible to transfer everything into this artificial system [35, junior]
So, on the one hand, the patient naturally talks to the physician and perhaps gives him information in certain situations that are simply not technically recorded. Well, and the big advantage is of course, the personal interaction, right? You see the patient, you see how he is, what the clinical impression is, that is not only laboratory values and that is good, that is somehow the overall picture [36, senior]
Well, I have to constantly trust people, that [they] have taken blood from the right patient, that the care says what I do, that the patients believe me that I somehow decide the right thing for them. And insofar in such a, in such a hospital all kinds of people have to trust each other all the time because you can't have an overview of anything [37, junior]
I am a counsellor who tries to get the best out of the patient and the final decision-making authority lies with the patient, because if he does not decide this himself, any therapy would probably be without success [38, senior]
[F]irst of all, I think, the medical team should decide among themselves what therapy options to propose to the patient in order to then propose these therapy options to the patient [39, junior]

I think it would be more difficult if you can't understand the decisions of the system, right? So, somehow the system has to be very transparent and has to list exactly what the decision it is making or the prediction it is making is based on. Because at the end of the day, it's not the system that talks to the patient, at least not yet, but we as physicians. And then I think we should know what these decisions are based on, so that we can communicate them openly and clearly [40, junior]
I still think that I would not use a system that I had to trust to such an extent that I could no longer understand the decision myself [41, junior]
I think the black box is totally okay, because I think I understood that the black box arrived at its results through an extremely large amount of data [42, junior]
A prospective study would of course also be important again in terms of validation. Or actually to test it again on a larger scale, where there are no biases in it [43, senior]
For artificial intelligence, I think you have to get used to it a little bit before you also can say this is how it's going to be. Especially in this area, right? [44, senior]
And in this respect, it also helps me when artificial intelligence gives me extra tips. But of course, I would try to convey it a little bit like that, depending on the individual patient. So it's not like: Ok, what do you want from me now, it's all there. In ten years they will be on dialysis [45, junior]
Just try to make the facts clear. So I'm assuming the general patient, who is not an expert in the field. Then there is always a need for certain simplification of the problem [46, senior]
[…] it immediately gives me a direction in which I think. I am or we are all somehow always so professionally suspicious and I have the one, also now because that was the first time to experience this application there, now not immediately a hundred per cent given myself to it. But it gives me immediately a trend and then relatively quickly if I already start to research then somehow, it actually always was confirmed. So I found as I said, it was then somehow a work relief [47, senior]
I think I always tried to look first myself and then to ask the AI again whether I had overlooked any of the points that the model found important and so on. And have quasi tried to get an unbiased view on it first and then again, again to let me support so to speak, because otherwise one becomes so lazy in thinking [48, senior]
And actually, it was often reflected what the system gave me and what I somehow found out. I would also claim that it was in the course that I was less suspicious […]. At the beginning like this: Okay, what is this? And then first of all this familiarisation

phase with the colour codes and the score. And it has then also always changed a little bit. But my considerations actually were confirmed after the first few times […]. Then I took the information with me, then went in search of it and quickly found myself confirmed [49, senior]
There was one case where I was annoyed afterwards that I didn't look at the proteinuria, because that was a big point in the AI for this case. And I thought to myself, okay, that's actually something that should have been looked at [50, senior]
So if I now, so if I was of the same opinion then I went along with it a bit, but if it was, completely, if I found it completely absurd, then I simply ignored it [51, junior]
[…] I think it's just, as I said before, an additional point that, as I said earlier, in this relatively quick and intuitive process, throws a moment of thought in between, even more when you might be in danger of overlooking something. But I think the decision-making process itself is relatively little influenced by that [52, junior]
I think I would always accept AI if the amount of data is just not analysable and capturable for me [53, senior]
[N]ot that it takes over my work, but that it helps me to record everything […] because I am no longer able to record all the data that is collected. It is difficult for me to look through a laboratory with 35 parameters and to look at every value and somehow not miss anything. And that helps me to somehow make a correct assessment or to point things out to myself [54, junior]
Above all, I think it is also about precise diagnosis. And above all, it is also about increasing efficiency. That you can practically reach the result more quickly, where you would perhaps also reach it without the system. […] [The system] does what I might be able to do in a much longer time, but it simply helps me to be more efficient by speeding up the whole process. And I don't have to do certain things in the end. So I think the clinical relevance is perhaps not at all that it now takes everything away from the physician in his clinical decision-making competence. It is […] a tool, which helps one to be more efficient in everyday life [55, senior]
[W]ell, that's also one of the dangers of it, that there is such a diffusion of responsibility in many things, right? […] And with the AI in particular, no one is to blame, because it has been trained with something. And nobody, that's just the way it is, there isn't even a human being who is responsible for the AI now. And in this respect I think it's very important that in the end it's still the physicians decision that stands at the end and is therefore also the one who bears the responsibility. And because an AI can't bear any responsibility [56, junior]

Of course, it would be a problem if I were to make a decision and the patient knew about the decision of the computer programme or the artificial intelligence and it was different from what I would recommend. I think that would be a problem [57, senior]
Of course, I wouldn't give the patients the feeling that I just give a few values into the computer and their therapy is completely decided by an artificial intelligence programme. But that it is an auxiliary instrument that I, as a human being, overlook as little as possible [58, senior]
[…] but you could perhaps shift them a bit emotionally. [...] And that would perhaps make life easier for the everyday work of a physician, because quite a few decisions can be depressing [59, senior]
So I think that is also a good process that, if you make a deviating recommendation now or come to a deviating result, that you just once again go on the way to look: Did I miss something? And I think that exactly is part of it [60, senior]
I mean, you can always take a special path, right? You just have to explain why you're doing it. And you're actually already doing that with the established tools. And if that is also an established tool and you still decide against it, then you just have to justify it. But I think that from the decision-making point of view it is something supportive but not dogmatic [61, senior]
I would want to set it up as a kind of prevention system, so that when something happens or something like that, you have this increased awareness or something, but would I integrate it into the direct decision-making process? I don’t know [62, junior]
If you see or fear therapy limitation or something and the system says, well, sure, we see it the same way, then you could give the patient another reason that supports your view of things. Maybe it would make it easier to make the decision that way then and then shed less light on whether other options were possible [63, senior]
That's what you should do, justify it to yourself. I think it's much more dangerous that you have to think about whether this system doesn't start to think for you at some point and you have to think about whether you're not resting too much on it and you're not going to be able to justify it to yourself [64, junior]
But if you say the AI is not supportive, it is already binding, then it becomes difficult, because maybe my therapy idea is different, so the artificial dictates. Then, I think it actually becomes difficult, because at some point you also come to the point where you simply no longer think sufficiently well about various things yourself. And then you quickly acquire this expert knowledge, which we all try to acquire somehow [65, junior]

For some of them maybe just an explanation why this parameter has maybe an influence, a bit more detailed explanation. Yes, most of the things I could somehow think of [why they are] important for completion or important for rejection, but there were a few where I didn't really understand the direct connection. So maybe a little bit more introduction to the individual ones [66, junior]
Yes, it would be good to know how it works, what [it] is based on, how it came about in the first place and on which factors. And that it is also explained on the pages that are now available for the cases, whereby for me, who has not yet dealt with it so much, it is still not easy. I also don't know how well one must understand it, but to understand what this [score] or this assessment is based on, that is quite difficult for me to understand. It probably makes sense to give more text and explanation or explanatory models or examples [67, senior]
I think if this AI had also been included [more choices] in the inside, then I would imagine it in the clinical setting. Namely, that I have to access another programme and so on. Now it seems very separate [68, junior]
It would be nice to have some more hints of a concrete nature. [...] For example, what were the most important indicators on the way to how the system now arrives at this? That you can look at it again, like in a timeline: Look, he's already had this event and he's just scrolling through it again. So that you can practically look it up again exactly by perhaps also looking at a timeline or at the criteria that were used. In the future it’s probably also, I don’t know, there’s a cross-link to interesting literature or: look, there were now 200 cases that are similarly published. You can then read them again. Here is the guideline and here are the therapy recommendations. Yes? […] That would be the next step, to think one step further [69, senior]
I think it's important that you can yourself let your prioritisation flow into it, that it's possible for you, that it can also be changed. That you don't somehow get a score where you had no possibility to influence it. That one simply makes use of the great computing power and also the better concentration ability of many variables, but says oneself: the variable is most important to me, then this one comes, then this one comes. And that you can possibly also say: okay, the result surprises me. Now I turn these two variables around again, because they are almost equally important [70, senior]
